# Development and Validation of Nutrition Literacy Questionnaire for the Chinese Elderly

**DOI:** 10.3390/nu14051005

**Published:** 2022-02-27

**Authors:** Sumiya Aihemaitijiang, Chen Ye, Mairepaiti Halimulati, Xiaojie Huang, Ruoyu Wang, Zhaofeng Zhang

**Affiliations:** Department of Nutrition & Food Hygiene, School of Public Health, Peking University Health Science Center, Haidian District, Beijing 100191, China; 1410606101@pku.edu.cn (S.A.); 1510306235@pku.edu.cn (C.Y.); 2011210145@bjmu.edu.cn (M.H.); 13161518166@163.com (X.H.); wry7582568@163.com (R.W.)

**Keywords:** the elderly, nutrition literacy, nutrition literacy questionnaire, multimorbidity

## Abstract

(1) Background: Improving nutrition literacy is crucial for maintaining a healthier state of the elderly to achieve healthy ageing. Therefore, it is necessary to develop a Nutrition Literacy Questionnaire for the Chinese Elderly (NLQ-E). (2) Methods: an NLQ-E was developed according to the core components of nutrition literacy for the elderly. Internal consistency, exploratory factor analysis (EFA) and confirmatory factor analysis (CFA) were used to validate the reliability and validity of the NLQ-E. A cross-sectional study of 1490 elderly people was used to analyze the application of the NLQ-E. (3) Results: The NLQ-E was constructed with 3 domains (knowledge and understanding, healthy lifestyle and dietary behavior and skill), with a total of 25 questions. The overall NLQ-E had acceptable reliability and validity (Cronbach’s α = 0.678, χ^2^/DF = 4.750, RMSEA = 0.045, PCFI = 0.776 and PNFI = 0.759). The average nutrition literacy score of the subjects in this cross-sectional study was 65.95 (65.95 ± 10.93). The OR between the nutrition literacy score and multimorbidity was 0.965 (95% CI: 0.954, 0.976); (4) Conclusions: We developed and validated the NLQ-E and found that the nutrition literacy level of the Chinese elderly was generally low. This study is of great value to improve the nutrition literacy of the elderly and effectively prevent nutrition-related chronic diseases and multimorbidity.

## 1. Introduction

Population aging is a common global trend and a major social change happening in China. According to the seventh population census of China in 2020, the population aged 60 and over was 264 million, accounting for 18.70% of the total population, of which the population aged 65 and over was 190 million, accounting for 13.50% of the total population. It is estimated that by 2030, the number of elderly people aged 65 and over will increase to approximately 240 million, of which the number of elderly people aged 80 and over will increase to more than 40 million. By 2050, the elderly population aged 80 and over will reach 100 million [1].

The change in demographic structure has led to changes in the Chinese epidemic and health status, and the main cause of death for people has changed from infectious diseases to chronic non-communicable diseases in China [2]. Although with the development of economy and the progress of medical technology, the average life expectancy of the elderly in China has been extended and the health level has been greatly improved, many elderly people still have irrational dietary structures due to the lack of nutritional knowledge and the influence of dietary culture and living habits. On the one hand, the consumption of vegetables, fruits, milk, beans, and bean products is insufficient, the intake of refined foods and high-fat and high-calorie foods has increased, and the intake of oil and salt is much higher than the recommended amount, resulting in overnutrition. On the other hand, the intake of dietary fiber and micronutrients is insufficient, especially vitamin A, vitamin D, calcium, and iron [3].

The imbalance of the dietary structure has induced the double burden of nutritional deficiency and overnutrition, which have exacerbated the occurrence and development of chronic non-communicable diseases, thus increasing the risk of multimorbidity and causing a great burden on themselves and society. A total of 48% of the elderly in China have poor nutritional status, the highest incidence of nutrition risk among the elderly in the Chinese community is 37%. The highest incidence of nutrition risk among the elderly in nursing homes is 60%, and the nutritional risk of hospitalized elderly patients is even as high as 65% [4]. Compared with the youth and middle aged, the functional organs of the elderly have different degrees of decline, such as decreased chewing and digestion ability, insufficient enzyme secretion, and decreased bone density, which greatly affect the digestion and absorption of the elderly [5], thereby further increasing the risk of nutrition-related chronic diseases and multimorbidity in the elderly.

Among many influencing factors, nutrition literacy is the most important factor affecting dietary intake. Nutrition literacy was defined by Zoellner [6] as an individual’s ability to obtain, process and understand basic nutrition information and use these abilities to make appropriate nutrition decisions and obtain nutrition services, based on health literacy which was first defined by the American National Library of Medicine [7] first defined in 2000. Kickbusch [8] emphasized that nutrition literacy should also include the ability to interpret nutrition policies, read nutrition labels, and choose healthier foods from different foods. Nutrition literacy includes nutritional knowledge, attitude towards nutritional health, nutritional health behavior and skill. Nutrition literacy plays an important role in the prevention and control of nutrition-related diseases such as diabetes, hypertension, cardiovascular and cerebrovascular diseases, and cancer [9,10,11,12]. People with sufficient nutrition literacy could choose healthier food [13]. However, the Chinese elderly had a low level of nutrition literacy [4]. In order to improve the nutritional status of the elderly, promote “healthy aging” and strengthen nutrition and health education, the State Council of China promulgated the National Nutrition Plan (2017–2030), which plans to increase the awareness rate of residents’ nutrition and health knowledge by 10%.

Therefore, scientific scales were needed to be established to evaluate nutrition literacy. At present, nutrition literacy evaluation tools include the Nutrition Literacy Assessment Instrument (NLit) [14], Nutritional Literacy Scale (NLS) [15,16], Evaluation Instrument of Nutrition Literacy on Adults (EINLA) [17] and Japanese Nutrition Literacy Scale [18]. However, these tools do not meet the needs of the Chinese elderly due to cultural differences and the physiological characteristics and eating habits of the elderly. The purpose of this study is to develop the Nutrition Literacy Questionnaire for the Chinese Elderly (NLQ-E) to screen the nutrition literacy of the elderly. This will provide a basis for formulating more targeted nutrition education strategies, improving the nutritional status of the elderly, preventing nutrition-related chronic diseases and multimorbidity of the elderly, and making nutrition education more standardized.

## 2. Materials and Methods

### 2.1. Construction of Questionnaire

The preparation of the nutrition literacy questionnaire mainly includes two stages:

Stage 1: The construction of the core components of nutrition literacy for the elderly.

The construction of the core components of nutrition literacy for the elderly was mainly divided into the following 3 aspects: 1: the framework system of the core components of nutrition literacy for the elderly was constructed by means of literature retrieval, group discussion and expert consultation; 2: the dimensions and core components was determined based on Dietary Guidelines for Chinese Residents (2016), Dietary Guidelines for the Elderly (2016), Auxiliary Reference Food Atlas of Retrospective Dietary Survey and the National Application for 2015 China Aging and Health Assessment; 3: hold an expert discussion meeting to discuss, analyze and modify the scientific, rationality, applicability and readability of nutrition literacy core components for Chinese elderly.

We have previously published a relevant article [19]. In this study, we used the updated core components of nutrition literacy of the elderly (Table 1).

Stage 2: Questionnaire development.

The Nutrition Literacy Questionnaire for the Chinese Elderly (NLQ-E) was established according to the core components of nutrition literacy for the Chinese elderly. The questionnaire consisted of two parts: the first part was the basic demographic information of the respondents, including age, gender, height, weight, education level and various disease histories; the second part was the Chinese elderly nutrition literacy assessment scale. The nutrition literacy part was divided into 3 aspects: knowledge and understanding, healthy lifestyle and dietary behavior, and skill, including 25 questions. The scale was designed using a 5-point Likert-type scoring method (“strongly disagree, disagree, do not know, agree, strongly agree”, “very inconformity, inconformity, average, compliance, very compliance”). The dimension of healthy lifestyle and dietary behavior involving food consumption frequency was set according to the recommended frequency in the Dietary Guidelines for the Chinese Elderly 2016, such as “0 days, 1–2 days, 3–4 days, 5–6 days and 7 days”. The skill dimension involves single choice questions. In the nutrition literacy part, 4 points were given for each question, totaling 100 points. The higher the score, the higher the nutrition literacy of the respondents.

### 2.2. Verification and Application of Questionnaire

#### 2.2.1. Data Collection and Samples

Convenient sampling was conducted in communities in Beijing, Hebei, Henan, Shandong and Tianjin Province, China. Questionnaires were distributed to elderly individuals aged 60 and above who have the ability to take care of themselves, excluding those who have cognitive and memory-related diseases, have no ability to read and express, and have trouble communicating with the investigators. Finally, a total of 1843 people were interviewed, 1490 valid questionnaires were collected, and the recovery rate was 80.85%. At the same time, written informed consent was obtained from all respondents.

The study protocol was approved by the Peking University Institutional Review Board (Beijing, China, approval number IRB00001052-19111) and conducted according to the Declaration of Helsinki and ethical guidelines. Written informed consent was obtained from all of the respondents. The privacy of elderly participants and the confidentiality of their personal information were protected.

#### 2.2.2. Reliability and Validity Tests

The reliability and validity of the questionnaire should be investigated by respondents who were 5–10 times the number of questions. The NLQ-E contains 3 domains and 25 questions, so the sample size was between 75 and 250. Approximately 17% of the subjects were randomly selected as the sample size for the reliability analysis [20].

Cronbach’α was used to evaluate internal consistency. Cronbach’s α > 0.7 meant that the reliability of the questionnaire was good (the minimum shall not be less than 0.6) [21].

Exploratory factor analysis (EFA) and confirmatory factor analysis (CFA) were used to analyze the structural validity of the questionnaire.

First, the Kaiser-Meyer-Olin (KMO) test (≥0.6) and Bartlett’s test of sphericity (*p* < 0.05) were used for suitability analysis to determine whether the questionnaire was suitable for EFA [22]. Then, principal component analysis and the maximum variance method were used to explore the factorial pattern (determined by the number of common factors, load value, variance of common factors and variance contribution rate of factors).

CFA was used to explore the consistency between the theoretical framework and the actual situation [23]. The ratio of the chi square value to degrees of freedom (χ^2^/DF), root mean square error of approximation (RMSEA) and thrift adjustment index (PCFI, PNFI) were used to evaluate the structural model fit of the questionnaire.

#### 2.2.3. Correlation Analysis between Nutrition Literacy Score and Multimorbidity

The incidence of multimorbidity in the elderly could be evaluated from the history of chronic diseases. In our study, the chronic diseases of the elderly were self-reported, including dyslipidemia (hyperlipidemia or hypolipidemia), diabetes or elevated blood sugar (including impaired glucose tolerance and elevated fasting blood sugar), hypertension, cancer and other malignant tumors (excluding mild skin cancer), chronic lung diseases such as bronchitis, emphysema, pulmonary heart disease (excluding tumor or cancer), liver diseases (except fatty liver, tumor or cancer), heart disease (such as myocardial infarction, coronary heart disease, angina pectoris, congestive heart failure and other heart diseases), stroke, kidney disease (excluding tumor or cancer), stomach disease or digestive system disease (excluding tumor or cancer), emotional and mental problems, memory related diseases (such as Alzheimer’s disease, brain atrophy, Parkinson’s disease), arthritis or rheumatism and asthma.

Subjects who suffered from the above two diseases at the same time were judged as multimorbidity [24]. Logistic regression analysis was used to explore the correlation between nutritional quality score and multiple incidence rate in the elderly.

#### 2.2.4. Statistical Analysis

All of the data should be reviewed by the investigator. After excluding the unqualified questionnaires, all questionnaires were entered through EpiData. The measurement data are expressed as the mean ± standard deviation, and Cronbach’s α and Spearman-Brown coefficients were used for reliability analysis. The structural validity analysis was expressed by χ^2^/DF, RMSEA, PCFI and PNFI. SPSS 27.0 statistical software was used for reliability and validity analysis, and amos22.0 statistical software was used for structural validity analysis. Moreover, *p* < 0.05 was used as the statistical index of significance test.

## 3. Results

### 3.1. Baseline Characteristics

A total of 1490 elderly participants participated in the study, 1176 female (78.93%) and 314 male (21.07%). The average age was 68.60 (68.60 ± 5.54) years, of which the maximum was 89 years. The average BMI was 25.23 (25.23 ± 4.51). Among them, 254 samples (17% of the subjects) were randomly selected for the analysis of the reliability and validity of the questionnaire, and the total samples were used for application analysis. The sociodemographic characteristics of the two research samples are shown in Table 2.

### 3.2. Reliability and Validity Tests

#### 3.2.1. Reliability

The overall NLQ-E had acceptable internal consistency (Cronbach’s α = 0.678, Spearman-Brown Coefficient = 0.711). The Cronbach’s α coefficients for the 3 domains of the NLQ-E (knowledge and understanding, healthy lifestyle and dietary behavior and skill) were 0.525, 0.730, and 0.367, respectively.

#### 3.2.2. Content Validity

The Pearson correlation coefficients between the scores of 3 domains (knowledge and understanding, healthy lifestyle and dietary behavior and skill) and the total score were 0.619, 0.746, and 0.755 (>0.5), which showed a strong correlation with the overall questionnaire (Table A1).

#### 3.2.3. Construct Validity

The results of KMO and Bartlett’s test of sphericity are shown in Table A2. The KMO value of the scale was 0.723 > 0.6, indicating that the NLQ-E was suitable for factor analysis.

Principal component analysis was conducted on 25 questions. A total of 10 common factors were extracted, and the cumulative variance contribution rate was 64.438% (Table A3). Through the factor load matrix of the data, it was that the 7th, 8th, 9th, and 10th factors had no obvious attribution (Table A4 and Table A5). Therefore, EFA extracted 6 factors.

On the whole, the overall model of the NLQ-E compiled in this study was well-suited. Table 3 shows the overall fitting coefficient of the NLQ-E. The χ^2^/DF was 4.750 (<5.0); RMSEA was 0.045 (<0.08); PCFI was 0.776 (>0.5); and PNFI was 0.759 (>0.5).

### 3.3. Status Quo of Nutrition Literacy of the Elderly

The average nutrition literacy score was 65.95 (65.95 ± 10.93), of which the highest score was 93 and the lowest score was 31. Taking 80 and above as the standard, 147 people (9.87%) were qualified (Table 4).

As shown in Table 4, the elderly with lower age, higher BMI and higher education level had significantly higher nutrition literacy (*p* < 0.05).

### 3.4. Factors Related to Nutrition Literacy

Multiple linear regression was performed to obtain the fitting model (Table 5, the model only leaved age and education level).

The partial regression coefficients of the variables included in the model were not 0 (*p*_age_ < 0.001; *p*_education level_ = 0.031 < 0.05). The increase in age was negatively correlated with the total score (B_age_ = −0.236 < 0), which meant that the older the age was, the lower the score, while the education level was positively correlated with the total score (B_education level_ = 0.888 > 0), which meant that the higher the education level was, the higher the score. The standardized beta: Beta_age_ = |−0.120| > |0.098|= Beta_education level_, indicating that age has a greater impact on the total score than education level.

In our survey, age and education level were related variables of nutrition literacy of the elderly. There was a negative correlation between age and nutrition literacy, while the level of education was positively correlated with nutrition literacy.

### 3.5. Correlation between Nutrition Literacy Score and Multimorbidity in the Elderly

The results of logistic regression analysis were shown in Table 6.

There were 1049 (70.40%) subjects with multimorbidity. After adjusting for age, gender, BMI and education level, the OR between the nutrition literacy score and multimorbidity was 0.965 (95% CI: 0.954, 0.976), indicating that every increase in nutrition literacy score would reduce the risk of multimorbidity by 3.5% (*p* < 0.001).

## 4. Discussion

This study for the first time constructed NLQ-E with 3 domains, including knowledge and understanding, healthy lifestyle and dietary behavior and skill, with a total of 25 questions. NLQ-E is a tool with acceptable reliability and validity to evaluate the nutrition literacy of the elderly.

The reliability of the scale is the stability and consistency of the results measured by the scale tool, that is, the degree of variation of the measurement results caused by random errors in the measurement process [25]. The higher the reliability, the more stable the measurement results of the scale. This study tested the reliability of NLQ-E through internal consistency reliability. Cronbach’s α coefficient is the most commonly used indicator of internal consistency reliability, which is generally considered as when the coefficient is greater than 0.7, the reliability of the scale is high, and the reliability between 0.6 and 0.7 is acceptable [26]. In our study, overall Cronbach’s α coefficient was 0.678, indicating that the overall reliability of the questionnaire was acceptable. The Cronbach’s α coefficients for the 3 domains of the NLQ-E (knowledge and understanding, healthy lifestyle and dietary behavior and skill) were 0.525, 0.730, and 0.367, respectively, indicating that the internal consistency of healthy lifestyle and dietary behavior domain was good. The reliability value of internal consistency depends on the number of items in the scale [27]. Since the questionnaire is aimed at the elderly, there are fewer questions in all. Domains of knowledge and understanding and skill have fewer questions, which may lead to low internal consistency. In addition, the small sample size and the overlapping content of different dimensions may be the reasons for the low α value.

The structural validity of the scale, also known as construction validity or conceptual validity, refers to the degree of consistency between the actual measurement and the theoretical conception model [28]. EFA and CFA were used to verify the structural validity of the scale. EFA mainly focuses on finding the correlation between topics, and CFA mainly focuses on the fitting ability between the pre-defined model and the actual data [29]. Seven common factors were extracted and named by principal component analysis: knowledge, understanding, dietary behavior, healthy lifestyle, cognitive skill, and operational skill. CFA of the Chinese elderly nutrition literacy assessment scale was carried out through the structural equation model. The results showed that: χ^2^/DF was 4.750 (<5.0), indicating that the adaptation was acceptable; RMSEA was 0.045 (<0.08), indicating that the adaptation was ideal, the PCFI was 0.776 (>0.5), indicating that the adaptation was acceptable, and the PNFI was 0.759 (>0.5), indicating that the adaptation was acceptable. It shows that the structural equation model of NLQ-E is successfully established, and the actual measurement basically aligns with the theoretical simulation.

NLQ-E was used to investigate 1490 elderly people over 60 in China. The total NLQ-E score of the respondents was 65.95 (65.95 ± 10.93), and the excellent rate of NL was 9.87% (score ≥ 80). In terms of knowledge and understanding, the understanding and judgment of “knowing that reasonable nutrition is the cornerstone of delaying aging and ensuring the health of the elderly” and “eating enough food to prevent nutritional deficiency” were poor, with low scores of 2.37 and 1.34, respectively, (full marks = 4). The recognition of “actively participating in the cooking process and eating with family or friends” was relatively high, with an average score of 3.24, indicating that the elderly had a good understanding of the dining environment. The core component “understanding food’s classification and nutritional value” was good commanded, with average scores above 3.2. The scores of further knowledge and understanding questions were more than 2.70, indicating that the dietary balance was not well mastered.

In terms of healthy lifestyle and dietary behavior, most of the subjects (1203, 80.74%) could eat 3 or more kinds of cereal, potato, and miscellaneous beans every day. Furthermore, 3/4 of the subjects (1135, 76.17%) could eat 4 or more kinds of vegetables and fruits every day. Half of the subjects (848, 56.91%) could eat 3 or more kinds of livestock, poultry, fish and eggs every day. Half of the subjects (892, 59.87%) could eat 2 or more kinds of milk, beans, and nuts every day. This result showed that food diversity was not achieved very well when choosing meals, which reducing the intake of nutrients and increasing the risk of multimorbidity. This might be related to the decrease in total food intake due to the decrease in chewing ability in the elderly. Therefore, food specially used for the elderly that are easy to digest and rich in nutrition should be vigorously developed. In addition, the low food diversity might also be related to the unwillingness or inability of elderly people living alone to cook multiple kinds of food. Therefore, it is suggested to establish more community restaurants to promote group dining and provide better choices for the elderly.

In terms of skill, except that the correct rate of egg weight estimation was 72.82% (1085 subjects were right), the correct rate of other skill questions was lower than 60.00%, indicating that their mastery was not particularly ideal, especially for the screening and selection of health products (the correct rate was 28.05%), as well as the reading and understanding of nutrition labels (the correct rate was 29.19%). This showed that the elderly had a poor grasp of basic skills. Age-related cognitive decline and lower education were the reasons for the poor understanding and low acceptance of the elderly. This suggests that we need to develop simple and easy-to-understand content to help the elderly learn and apply nutrition knowledge and skills quickly in the process of nutrition education.

Our study showed that nutrition literacy level of the elderly with the older age, the lower BMI and the lower education level was lower. In addition, our study found that nutrition literacy level was negatively correlated with multimorbidity in the elderly. This is because low nutrition literacy level would directly lead to unhealthy dietary patterns and unhealthier nutritional status, which were considered to be associated with increased incidence rate of various health diseases [30]. It is suggested that improving the nutrition literacy of the elderly could be used as a preventive strategy for multimorbidity.

The limitations of this study are the use of convenient sampling rather than random sampling when issuing the questionnaire, and the higher proportion of females versus male respondents. Some indicators in the process of questionnaire verification may only reach an acceptable level and can be improved in subsequent research. The questionnaire only involves intake frequency, and there is no accurate intake, which also needs further revision.

## 5. Conclusions

In conclusion, we had developed and validated the NLQ-E for people over 60 years old and found that the nutrition literacy level of the Chinese elderly was generally low. Nutrition literacy level was negatively correlated with the occurrence of multimorbidity in the elderly. Scientific and simple nutrition intervention strategies should be formulated to improve the nutritional status of the elderly and standardize nutrition education.

## Figures and Tables

**Table 1:** Core components of nutrition literacy of the elderly.

Domain	Dimension	Component
Knowledge and understanding	Knowledge	1. Understanding food’s classification and nutritional value.
	Understanding	2. Knowing that reasonable nutrition is the cornerstone of delaying aging and ensuring the health of the elderly.
		3. Eating enough food to prevent nutritional deficiency.
		4. Actively preventing muscle attenuation and osteoporosis to reduce the incidence of chronic diseases.
		5. Constantly monitoring weight changes and maintaining appropriate weight.
		6. Actively participating in the cooking process and eating with family or friends.
Healthy lifestyle and dietary behavior	Dietary behavior	7. Eating a variety of foods, with an average of more than 12 kinds of food per day.
		8. Eating soft food, chewing carefully and swallowing slowly, eating a small amount of more meals, eating regularly and having a good breakfast.
		9. Increasing the intake of whole grains and miscellaneous beans.
		10. Eating enough high-quality protein and enough fish, poultry, eggs and meat.
		11. Eating enough vegetables and fruits.
		12. Reasonably selecting high calcium food to ensure milk product and bean product intake.
		13. Less salt, less oil and less sugar.
		14. Take the initiative to drink enough water, especially warm boiled water.
	Healthy lifestyle	15. Actively participating in outdoor activities and doing resistance exercise appropriately.
Skill	Cognitive skill	16. Paying attention to nutrition and health information, identifying and applying correct information.
	Operational skill	17. Learning to estimate the amount of food and mix food reasonably.
		18. Learning to read food nutrition labels and choose food reasonably.
		19. Making rational use of nutritious fortified foods or nutrient supplements and with the help of nutritionists and doctors.
		20. Paying attention to food hygiene and learning to make rational use of surplus meals.

**Table 2:** Demographic characteristics of participants, *n* (%).

Characteristics	Total (*N* = 1490)	Reliability and Validity Tests (*N* = 254)
Age (mean ± SD)	68.60 ± 5.543	68.18 ± 5.259
BMI (mean ± SD)	25.23 ± 4.508	25.45 ± 4.458
Gender		
Male	314 (21.07)	52 (20.47)
Female	1176 (78.93)	202 (79.53)
Education level		
Illiteracy	43 (2.89)	11 (4.33)
Did not finish primary school	160 (10.74)	25 (9.84)
Grad from primary	216 (14.50)	40 (15.75)
Lower middle school degree	571 (38.32)	97 (38.19)
Upper middle school degree	393 (26.37)	65 (25.59)
Technical or vocational degree	83 (5.57)	14 (5.51)
Bachelor degree or above	24 (1.61)	2 (0.79)

**Table 3:** Overall fitting coefficient table.

	χ^2^/df	RMSEA	PCFI	PNFI
Ideal value	≤3.0	≤0.08	1.0	1.0
Acceptable value	≤5.0	≤0.1	>0.50	>0.50
Result	4.740	0.045	0.776	0.759

RMSEA: root mean square error of approximation; PCFI, PNFI: thrift adjustment index.

**Table 4:** Distribution of nutrition literacy in the elderly (*n* = 1490, mean ± SD).

Variables (Subjects)	Total (100′)	Knowledge and Understanding (36′)	Healthy Lifestyle and Dietary Behavior (36′)	Skill (28′)
Total	65.95 ± 10.93	24.27 ± 4.30	28.01 ± 5.93	13.67 ± 6.03
Gender				
Male (314)	65.64 ± 11.78	24.50 ± 4.75	28.27 ± 5.86	12.87 ± 6.36
Female (1176)	66.03 ± 10.69	24.20 ± 4.17	27.94 ± 5.95	13.89 ± 5.92
Age				
≥60 & <70 (912)	66.92 ± 11.05	24.33 ± 4.26	28.49 ± 5.99	14.11 ± 5.96
≥70 & <80 (500)	64.97 ± 10.45 ^a^	24.24 ± 4.33	27.54 ± 5.82 ^a^	13.19 ± 6.00 ^a^
≥80 (78)	60.77 ± 10.54 ^a^	23.65 ± 4.59	25.42 ± 4.96 ^a^	11.69 ± 6.41 ^a^
BMI				
<18.5 (32)	59.56 ± 9.85	22.19 ± 4.53	26.00 ± 4.76	11.38 ± 6.02
≥18.5 & <24 (557)	65.25 ± 11.41 ^b^	24.39 ± 4.72 ^b^	27.65 ± 6.04	13.21 ± 6.19
≥24 (901)	66.94 ± 11.40 ^b^	24.27 ± 4.74 ^b^	27.56 ± 5.99 ^b^	13.11 ± 6.19 ^b^
Education level				
Illiteracy (43)	63.44 ± 9.97	24.26 ± 5.32	26.72 ± 5.05	12.47 ± 6.25
Did not finish primary school (160)	64.01 ± 10.51	25.12 ± 4.47	26.66 ± 5.74	12.23 ± 5.87
Grad from primary (216)	63.93 ± 10.24	23.88 ± 4.44	26.51 ± 5.69	13.54 ± 5.91
Lower middle school degree (571)	66.48 ± 11.34	24.04 ± 4.33	28.61 ± 6.00	13.84 ± 6.08
Upper middle school degree (393)	66.74 ± 10.68	24.39 ± 3.96	28.36 ± 5.82	13.98 ± 6.09
Technical or vocational degree (83)	67.45 ± 11.01 ^c^	24.56 ± 4.09	28.53 ± 5.72	14.46 ± 5.72
Bachelor degree or above (24)	70.83 ± 10.75 ^c^	24.79 ± 4.69	31.04 ± 3.95^c^	15.00 ± 5.31

^a^: Compared with the elderly aged 60–70, the score of nutrition literacy was different (*p* < 0.05). ^b^: Compared with the elderly with BMI less than 18.5, the score of nutrition literacy was different (*p* < 0.05). ^c^: Compared with the illiterate elderly, the score of nutrition literacy was different (*p* < 0.05).

**Table 5:** Estimate of partial regression.

Variables	Non-Standardized Coefficient		Standardized Coefficient	t	*p*
	B	SE	Beta		
(Constant)	78.578	3.762		20.885	<0.001 *
Age	−0.236	0.051	−0.120	−4.619	<0.001 *
Education level	0.888	0.233	0.098	3.802	<0.001 *

*: *p* < 0.05.

**Table 6:** Logistic regression analysis between nutrition literacy score and multimorbidity.

	OR	95% CI	*p*
Total score	0.960	0.950, 0.971	<0.001 *
Adjusted			
Age	1.029	1.006, 1.052	
Gender	0.198	0.894, 1.606	0.2266
BMI	0.988	0.963, 1.013	0.3374
Education level			
Illiteracy: Bachelor degree or above	9.509	2.252, 40.154	0.0020 *
Did not finish primary school: Bachelor degree or above	3.213	1.282, 8.052	0.0128 *
Grad from primary: Bachelor degree or above	2.849	1.167, 6.952	0.0215 *
Lower middle school degree: Bachelor degree or above	2.038	0.873, 4.760	0.0999
Upper middle school degree: Bachelor degree or above	1.434	0.609, 3.374	0.4093
Technical or vocational degree: Bachelor degree or above	1.239	0.483, 3.176	0.6556
Total score	0.965	0.954, 0.976	<0.001 *

*: *p* < 0.05.

## Data Availability

The data presented in this study are available from the corresponding author on request.

## Appendix A

**Table A1:** Correlation coefficients between the scores of three domains and the total score.

		Knowledge and Understanding	Healthy Lifestyle and Dietary Behavior	Skill	Total
Knowledge and understanding	Pearson Correlation	1	0.257 **	0.236 **	0.619 **
	p (2-tailed)		<0.001	<0.001	<0.001
	N	254	254	254	254
Healthy lifestyle and dietary behavior	Pearson Correlation	0.257 **	1	0.282 **	0.746 **
	p (2-tailed)	0.000		<0.001	<0.001
	N	254	254	254	254
Skill	Pearson Correlation	0.236 **	0.282 **	1	0.755 **
	p (2-tailed)	<0.001	<0.001		<0.001
	N	254	254	254	254
Total	Pearson Correlation	0.619 **	0.746 **	0.755 **	1
	p (2-tailed)	<0.001	<0.001	<0.001	
	N	254	254	254	254

**. Correlation is significant at the 0.01 level (2-tailed).

**Table A2:** KMO and Bartlett’s Test.

Kaiser-Meyer-Olkin Measure of Sampling Adequacy.		0.723
Bartlett’s Test of Sphericity	Approx. Chi-Square	2784.464
	df	465
	p	<0.001

**Table A3:** Total Variance Explained.

Component	Total	% of Variance	Cumulative %
1	3.791	12.230	12.230
2	2.742	8.884	21.074
3	2.340	7.549	28.623
4	1.885	6.080	34.703
5	1.800	5.807	40.510
6	1.603	5.170	45.680
7	1.575	5.079	50.759
8	1.561	5.035	55.794
9	1.346	4.343	60.137
10	1.33	4.301	64.438

**Table A4:** Rotated Component Matrix ^a^.

Question	Component									
	1	2	3	4	5	6	7	8	9	10
1	0.032	0.239	−0.073	0.097	0.303	0.671	0.106	0.119	−0.079	0.201
2	0.053	−0.044	−0.018	−0.119	−0.147	0.822	−0.100	0.045	−0.022	−0.079
3	−0.015	−0.020	0.022	0.096	0.539	0.426	0.312	−0.090	0.151	−0.267
4	−0.051	0.082	−0.113	0.588	0.095	−0.323	0.168	−0.145	−0.122	0.118
5	0.124	0.000	0.102	−0.100	0.725	0.013	−0.048	−0.027	0.022	0.284
6	−0.050	0.115	0.040	0.851	−0.079	−0.007	0.009	0.044	0.029	0.056
7	0.031	0.033	−0.021	0.798	0.003	0.099	−0.077	0.121	0.090	−0.005
8	0.001	0.098	−0.048	0.026	0.746	−0.050	−0.016	0.044	−0.027	−0.013
9-1	0.895	0.112	0.080	−0.014	0.021	0.056	0.019	0.023	0.045	0.005
9-2	0.940	0.022	0.078	−0.073	0.051	−0.013	0.098	0.011	0.032	−0.006
9-3	0.958	0.015	0.042	0.007	0.038	0.010	0.086	0.014	0.054	0.025
9-4	0.942	0.025	0.022	0.019	0.001	0.034	0.078	0.011	0.036	0.060
10-1	0.150	0.160	0.628	0.168	−0.118	0.166	−0.096	−0.033	−0.218	−0.019
10-2	0.197	−0.037	0.769	−0.088	−0.035	−0.037	0.030	−0.010	0.003	−0.067
10-3	−0.097	0.247	0.725	−0.112	0.000	−0.111	0.112	0.087	0.088	0.119
10-4	−0.014	0.096	0.802	0.021	0.189	−0.044	0.138	0.053	0.117	0.105
11	0.326	0.379	0.034	0.025	0.195	−0.049	0.140	0.001	0.025	−0.368
12	0.102	0.595	0.082	−0.012	−0.117	−0.017	−0.058	0.068	−0.019	0.265
13	0.104	0.662	0.183	−0.035	0.007	−0.090	0.216	0.057	−0.105	−0.168
14	−0.035	0.712	0.074	0.131	0.094	0.080	0.056	−0.095	0.133	0.082
15	−0.006	0.650	0.083	0.164	0.151	0.103	−0.053	0.000	−0.062	−0.077
16	0.000	0.557	0.085	0.024	−0.117	−0.010	0.526	−0.104	0.118	−0.010
17	0.082	0.310	−0.125	0.036	−0.065	−0.035	−0.047	0.385	0.459	−0.087
18	0.061	0.454	−0.083	−0.084	0.114	0.030	0.050	0.174	0.506	0.153
19	0.112	0.048	0.162	−0.013	0.232	−0.234	0.237	0.020	0.265	0.464
20	−0.004	−0.002	0.073	0.007	−0.067	0.075	0.174	0.757	0.049	0.063
21	0.026	−0.029	0.028	0.065	0.087	0.037	−0.073	0.828	−0.003	−0.020
22	0.033	0.060	0.031	0.125	0.094	0.046	0.085	0.008	−0.029	0.728
23	0.147	−0.047	0.093	0.000	−0.110	0.108	0.668	0.041	0.044	0.230
24	−0.078	0.124	−0.099	−0.068	0.004	0.028	0.004	0.057	−0.785	−0.002
25	0.156	0.194	0.050	0.012	0.207	−0.157	0.672	0.117	−0.102	−0.057

Extraction Method: Principal Component Analysis. Rotation Method: Varimax with Kaiser Normalization. ^a^. Rotation converged in 8 iterations.

**Table A5:** Rotated Component Matrix ^a^.

Question	Component					
	1	2	3	4	5	6
1	0.010	0.288	−0.180	0.416	−0.133	0.406
2	0.036	0.028	−0.211	−0.074	−0.484	0.422
3	0.010	0.161	−0.128	0.547	−0.145	0.105
4	−0.042	0.119	−0.090	0.060	0.674	−0.217
5	0.103	−0.057	0.122	0.717	−0.022	−0.037
6	−0.057	0.178	−0.060	−0.137	0.732	0.209
7	0.019	0.091	−0.151	−0.077	0.625	0.327
8	−0.017	0.085	−0.075	0.638	0.031	−0.033
9-1	0.886	0.128	0.067	0.021	−0.044	0.076
9-2	0.942	0.050	0.095	0.062	−0.061	0.005
9-3	0.960	0.043	0.053	0.057	0.009	0.042
9-4	0.942	0.048	0.034	0.033	0.019	0.054
10-1	0.116	0.207	0.478	−0.214	−0.021	0.076
10-2	0.192	0.015	0.713	−0.093	−0.140	−0.017
10-3	−0.102	0.244	0.765	0.025	−0.041	0.045
10-4	−0.015	0.137	0.789	0.190	0.031	0.068
11	0.327	0.457	−0.028	0.084	−0.062	−0.049
12	0.071	0.499	0.144	−0.058	0.071	0.094
13	0.096	0.696	0.191	−0.030	−0.042	−0.046
14	−0.052	0.701	0.055	0.128	0.118	0.041
15	−0.042	0.647	−0.005	0.067	0.053	0.078
16	0.036	0.656	0.162	0.045	0.087	−0.093
17	0.086	0.259	−0.060	−0.038	0.082	0.428
18	0.067	0.389	0.021	0.245	0.036	0.283
19	0.134	−0.005	0.352	0.411	0.271	−0.028
20	0.002	−0.017	0.179	0.008	0.028	0.639
21	0.006	−0.088	0.078	0.032	0.037	0.689
22	0.023	−0.033	0.169	0.312	0.313	0.085
23	0.206	0.078	0.228	0.172	0.094	0.040
24	−0.116	0.111	−0.134	−0.098	−0.141	−0.170
25	0.202	0.322	0.168	0.309	0.116	−0.101

Extraction Method: Principal Component Analysis. Rotation Method: Varimax with Kaiser Normalization. ^a^. Rotation converged in 8 iterations.
